# Estimating the accuracy of muscle response testing: two randomised-order blinded studies

**DOI:** 10.1186/s12906-016-1416-2

**Published:** 2016-11-30

**Authors:** Anne M. Jensen, Richard J. Stevens, Amanda J. Burls

**Affiliations:** 1 Department of Primary Care Health Sciences, University of Oxford, Oxford, UK; 2 Department for Continuing Education, University of Oxford, Oxford, UK; 3 School of Health Sciences, City University London, London, UK

**Keywords:** Sensitivity, Specificity, Muscle weakness, Lie detection, Kinesiology

## Abstract

**Background:**

Manual muscle testing (MMT) is a non-invasive assessment tool used by a variety of health care providers to evaluate neuromusculoskeletal integrity, and muscular strength in particular. In one form of MMT called muscle response testing (MRT), muscles are said to be tested, not to evaluate muscular strength, but neural control. One established, but insufficiently validated, application of MRT is to assess a patient’s response to semantic stimuli (e.g. spoken lies) during a therapy session. Our primary aim was to estimate the accuracy of MRT to distinguish false from true spoken statements, in randomised and blinded experiments. A secondary aim was to compare MRT accuracy to the accuracy when practitioners used only their intuition to differentiate false from true spoken statements.

**Methods:**

Two prospective studies of diagnostic test accuracy using MRT to detect lies are presented. A true positive MRT test was one that resulted in a subjective weakening of the muscle following a lie, and a true negative was one that did not result in a subjective weakening of the muscle following a truth. Experiment 2 replicated Experiment 1 using a simplified methodology. In Experiment 1, 48 practitioners were paired with 48 MRT-naïve test patients, forming unique practitioner-test patient pairs. Practitioners were enrolled with any amount of MRT experience. In Experiment 2, 20 unique pairs were enrolled, with test patients being a mix of MRT-naïve and not-MRT-naïve. The primary index test was MRT. A secondary index test was also enacted in which the practitioners made intuitive guesses (“intuition”), without using MRT. The actual verity of the spoken statement was compared to the outcome of both index tests (MRT and Intuition) and their mean overall fractions correct were calculated and reported as mean accuracies.

**Results:**

In Experiment 1, MRT accuracy, 0.659 (95% CI 0.623 - 0.695), was found to be significantly different (p < 0.01) from intuition accuracy, 0.474 (95% CI 0.449 - 0.500), and also from the likelihood of chance (0.500; p < 0.01). Experiment 2 replicated the findings of Experiment 1. Testing for various factors that may have influenced MRT accuracy failed to detect any correlations.

**Conclusions:**

MRT has repeatedly demonstrated significant accuracy for distinguishing lies from truths, compared to both intuition and chance. The primary limitation of this study is its lack of generalisability to other applications of MRT and to MMT.

**Study registration:**

The Australian New Zealand Clinical Trials Registry (ANZCTR; www.anzctr.org.au; ID # ACTRN12609000455268, and US-based ClinicalTrials.gov (ID # NCT01066312).

**Electronic supplementary material:**

The online version of this article (doi:10.1186/s12906-016-1416-2) contains supplementary material, which is available to authorized users.

## Abstrakt

**Ziele:**

Abschätzung der Treffgenauigkeit von kinesiologischem, manuellem Muskelabtasten (Muskelabtasten im kinesiologischen Stil) (MRT) zum Unterscheiden zwischen Lügen und Wahrheit in gesprochenen Aussagen.

**Studiendesign:**

Zwei prospektive Studien über diagnostische Treffgenauigkeit von MRT zur Entdeckung von Lügen werden präsentiert. Eine tatsächlich positives MRT Testresultat liegt vor, wenn eine Muskelabschwächung resultierte und ein tatsächlich negatives MRT bei keiner Muskelabschwächung. Versuch 2 wiederholte Versuch 1 unter Anwendung einer vereinfachten Methodik.

**Durchführungsort:**

Private Praxen in Grossbritannien und Vereinigte Staaten, mit einem Fundus an Testpatienten (TPs) aus der lokalen Gesellschaft.

**Teilnehmende:**

Im Versuch 1, 48 Fachausübende wurden mit 48 MRT unbefangenen TPs verkuppelt und formten damit einmalige Paare von Fachausübenden-TP („Paare“). Fachausübende mit irgend welcher MRT Erfahrung wurden zugelassen. Im Versuch 2 wurden 20 einmalige Paare zugelassen, wobei die TPs aus einem Mix von MRT Unbefangenen und Befangenen bestanden.

**Testindex:**

Der prmäre Testindex war MRT. Ein sekundärer Testindex wurde ebenfalls durchgeführt, bei welchem die Fachausübenden intuitive Vermutungen („Intuition“), ohne Anwendung von MRT., anstellten.

**Angewendeter Standardtest (Referenzstandardtest):**

Der effektive Wahrheitsgehalt der gesprochenen Aussage wurde verglichen mit dem Resultat des Textindex und das Gesamtmittel des korrekten. Aussageanteils wurde berechnet und als durchschnittliche Treffgenauigkeit ausgewiesen.

**Resultate:**

Im Versuch 1, MRT Treffgenauigkeit, 0.659 (95% CI 0.623 - 0.695), wurde als signifikant unterschiedlich (p < 0.01) von intuitiver Treffgenauigkeit, 0.474 (95% CI 0.449 - 0.500),und wie auch von der Zufallswahrscheinlichkeit (0.500; p < 0.01) identifiziert. Experiment 2 reproduzierte die Ergebnisse des Versuchs 1. Es konnten keine Korrelationen von anderen Faktoren identifiziert werden, welche die MRT Treffgenauigkeit hätten beeinflussen können.

**Fazit:**

MRT hat wiederholt signifikante Treffgenauigkeit zum Unterscheiden zwischen Lügen und Wahrheit gezeigt im Vergleich zu Intuition und Zufall. Die primäre Einschränkung dieser Studie liegt in Mangel der Uebertragbarkeit auf andere Anwendungsgebiete der MRT.

## Background

Manual muscle testing (MMT) is a non-invasive assessment tool used by a variety of health care providers, including physiotherapists, chiropractors, osteopaths and medical doctors, to evaluate neuromusculoskeletal integrity for a variety of purposes [1, 2]. One form of MMT, muscle response testing (MRT), in which muscles are tested, not to evaluate muscular strength, but neural control, emerged following work in the 1970s and1980s by Goodheart and others [3, 4]. Because MRT is estimated to be used by over 1 million people worldwide [5], assessing its validity is necessary. Distinguishing MRT from other types of manual muscle testing, typically only one muscle is used for testing, and is tested repeatedly, to detect the presence of potential target conditions, such as low back pain [6]. simple phobia [7, 8], and food allergies [9].

One established application of MRT is to assess a patient’s response to semantic stimuli (e.g. spoken statements) during a therapy session [3, 10, 11]. The semantic stimulus can be spoken by the patient or the practitioner, and practitioners monitor a patient’s muscular resistance to pressure they apply at the same time as they, or the patients, speak statements. A previous study of 89 test subjects showed that following the speaking of true statements, a muscle resists significantly more force compared to after speaking false statements [12]. However, key details were not reported, such as the number of practitioners taking part and, in particular, the level of blinding. A protocol was published in 2009 for a randomised controlled trial of such a therapy which uses MRT, but trial results have not yet reached journal publication [13].

Our primary aim was to estimate the accuracy of MRT to distinguish false from true spoken statements, in randomised and blinded experiments. A secondary aim was to compare MRT accuracy to the accuracy when practitioners used only their intuition to differentiate false from true spoken statements.

## Methods

**Fig. 1:**
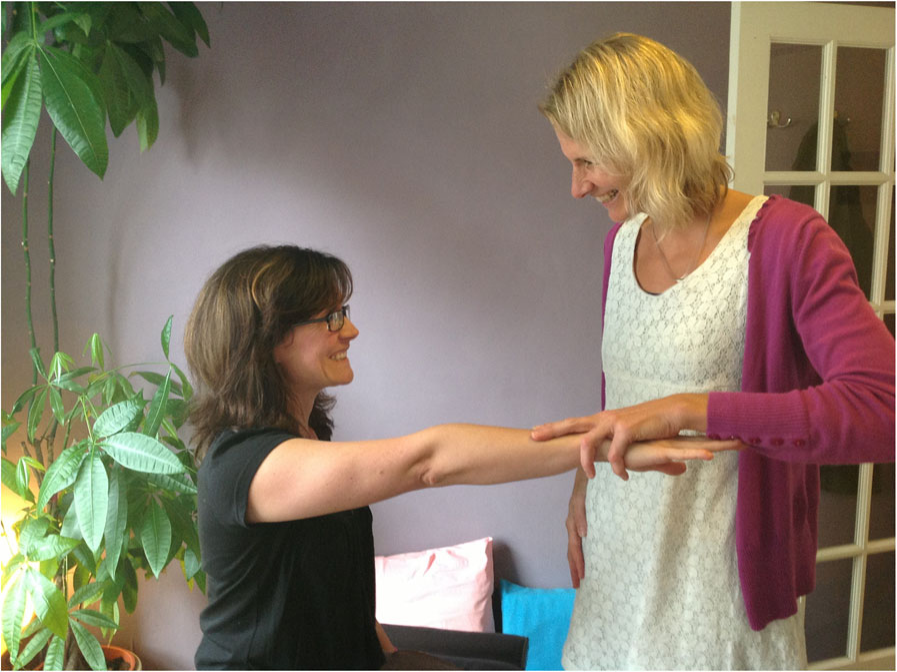
An example of a practitioner performing MRT using a patient's right deltoid muscle


These studies were prospective studies of diagnostic test accuracy, were registered with two clinical trials registries: the Australian New Zealand Clinical Trials Registry (ANZCTR; www.anzctr.org.au; ID # ACTRN12609000455268), and US-based ClinicalTrials.gov (ID # NCT01066312); and received ethics committee approval to collect data in the United Kingdom and the United States. For data collection in the United Kingdom ethics approval was granted from the Oxford Tropical Research Ethics Committee (OxTREC Reference Numbers 34-09 and 41-10), and for data collection in the United States, from the Parker University Institutional Review Board (Approval Numbers R09-09 and R15-10). Consent to publish was obtained from everyone featured in both Fig. 1 and the Additional file videos 1 and 2. Written informed consent was obtained from all participants, and all other tenets of the Declaration of Helsinki were upheld. In addition, these studies are reported in accordance with the Standards for the Reporting of Diagnostic Test Accuracy Studies (STARD) guidelines [14–16]. For STARD Checklists, see Additional file 3: Table S6 and Additional file 4: Table S7.

Additional file 1: (WMV 4350 kb)
Additional file 2: (WMV 6250 kb)


The paradigm tested in this study was one in common use in clinical practice: lying (i.e. speaking a false statement) results in a weak MRT response, whereas telling the truth (i.e. speaking a true statement) results in a strong response. We treat a weak muscle response as a positive index test for diagnosing a lie. If the muscle stayed strong, it was considered a negative test result for deceit.

For comparison, a second index test was also evaluated: intuition. During this phase, practitioners were asked to use their intuition (or to “guess”) in order to ascertain the truthfulness – without using MRT. Because deceit is known to be accompanied by various physiological changes [17–19], practitioners were asked to use only their senses to detect deceit: sight (e.g. by observing body language and facial expressions), hearing (e.g. changes in voice qualities) and touch (e.g. changes in skin temperature).

In both experiments, four blocks of 10 MRTs alternated with 4 blocks of 10 intuitions, always beginning with a MRT block. Practitioners alone determined the outcome of the MRTs and intuitions, and they themselves entered the results into a computer using a keyboard.

### Experiment 1

#### Participants

Two groups of participants were recruited: (1) Healthcare practitioners (“practitioners”; *n* = 48) who routinely use MRT in practice, and (2) Test Patients (“TPs”; *n* = 48) who were naïve to MRT. Each practitioner was paired with a unique TP and together they formed a unique testing pair (“pair”; hence, *n* = 48 unique pairs). Recruitment was by direct contact (via email or telephone), social media and word of mouth. Any volunteer was eligible if he or she was aged 18–65 years, had fully functioning and painfree upper extremities, and was fluent in English. Volunteers were excluded if they were blind, deaf or mute. TPs were also paired with practitioners they did not know. All practitioners who wished to participate and met the inclusion criteria were enrolled, regardless of their profession, MRT technique(s) used, or extent of MRT expertise or experience. No practitioner’s muscle testing ability was assessed in any way prior to enrolment.

#### The Primary Index Test: MRT

During a MRT, an external force is applied to a body appendage and resisted by a particular muscle. At first the patient holds a specific joint in a fixed position, usually in partial flexion. The practitioner then applies pressure, usually into extension, as the patient resists this pressure using an isometric contraction. For example, the practitioner may ask the patient to hold his shoulder (i.e. the glenohumeral joint) in 90° flexion, palm facing down, while he tests the anterior deltoid (see Fig. 1). The practitioner then subjectively determines if the muscle went “weak” or stayed “strong.”

Practitioners may vary in the amount of pressure applied and location of the practitioner’s hand [20]. The location is routinely on the distal forearm of the patient, just proximal to the wrist joint, but for the purposes of this study practitioners were instructed to follow their usual clinical practice in muscle testing.

#### Test methods

TPs spoke 40 statements of mixed verity as follows. They viewed pictures on a computer screen placed out of view of the practitioners. While viewing a picture selected at random by computer, the TPs were given instructions by computerised voice via an earpiece inaudible to the practitioners. Instructions took the form, “Say, ‘I see a ________.’” The verity of the statements (that is, whether the instructed statement was chosen to match the picture on screen) were randomly allocated by software (DirectRT Research Software, Empirisoft Corporation, New York, NY), with overall prevalence of lies set to be 50 ± 3%. The practitioner also viewed a computer screen and was randomly shown either the same picture as the TP (i.e. not blind) or a blank black screen (i.e. blind). Participants were blind to study aims and were not informed of the proportions of True/False statements or Blind/Not Blind cases. Pictures of neutral valence (i.e. emotionally neutral) were chosen from the International Affective Picture System (IAPS; National Institute of Mental Health Center for Emotion and Attention, University of Florida, Gainesville, FL) [21] and paired with neutral words selected from the Affective Norms for English Words (ANEW; National Institute of Mental Health Center for Emotion and Attention, University of Florida, Gainesville, FL) [22].

Following each statement spoken by the TP, the practitioner was asked to estimate the verity of the statement: ten times using MRT, followed by ten times using intuition alone, and alternating in blocks of ten thereafter (see Additional file 5: Figure S1). The practitioner entered their estimate for each statement by single key press on a keyboard connected to the study computer, which automatically collated results. Practitioners and TPs were allowed a short period to familiarise themselves with study layout and procedures before beginning, and the principal investigator was present in the room during data collection but did not take part.

Participants were asked to complete two short questionnaires, one before testing started and one after testing was completed. The TP Pre-testing Questionnaire included questions about age, gender, handedness, MRT experience, and levels of confidence in MRT, in their practitioner, and their practitioner’s MRT. The practitioner pre-testing questionnaire included questions about age, gender, handedness, type of practitioner, years in practice, years of MRT experience, self-rated MRT expertise, specific MRT techniques used, and levels of confidence in MRT in general and their own MRT ability. Levels of confidence were measured using a 10 cm Visual Analogue Scale (VAS) with the left end marked “None” and the right end marked with “Complete Confidence.” All participants were asked to use a “|” to mark the VAS, which was subsequently assigned a score out of 10. Practitioners were asked to rate their own MRT expertise using a 5-point Likert scale from 0 (None) to 4 (Expert). We combined categories 1 and 2 of self-reported expertise due to low numbers (e.g. *n* = 1 whose reported their expertise was at level 1). Lengths of time, such as ages and years in practice, were kept as continuous variables, while other variables, such as gender, profession, and MRT techniques used, were kept as categorical variables.

In the Post-testing Questionnaire, participants were again asked to rate the same levels of confidence. In addition, in the Post-testing Questionnaire, TPs were asked to make open-ended comments about anything they noticed during the MRT, in order to establish if they deduced the paradigm under investigation (i.e. lies result in a “weak” MRT response), so that response bias can be measured [23, 24]. As a means of fidelity assurance during this experiment, the principle investigator (AJ) was present during all testing and assessment.

### Experiment 2

Following completion and analysis of Experiment 1, a replication experiment was designed as follows.

#### Participants

Participants were enrolled in a similar way to Experiment 1; however, the sample size was reduced to 20 pairs, and some non-MRT-naïve TPs were recruited and enrolled. Also included were some pairs that were acquainted with each other.

#### Test methods

**Fig. 2:**
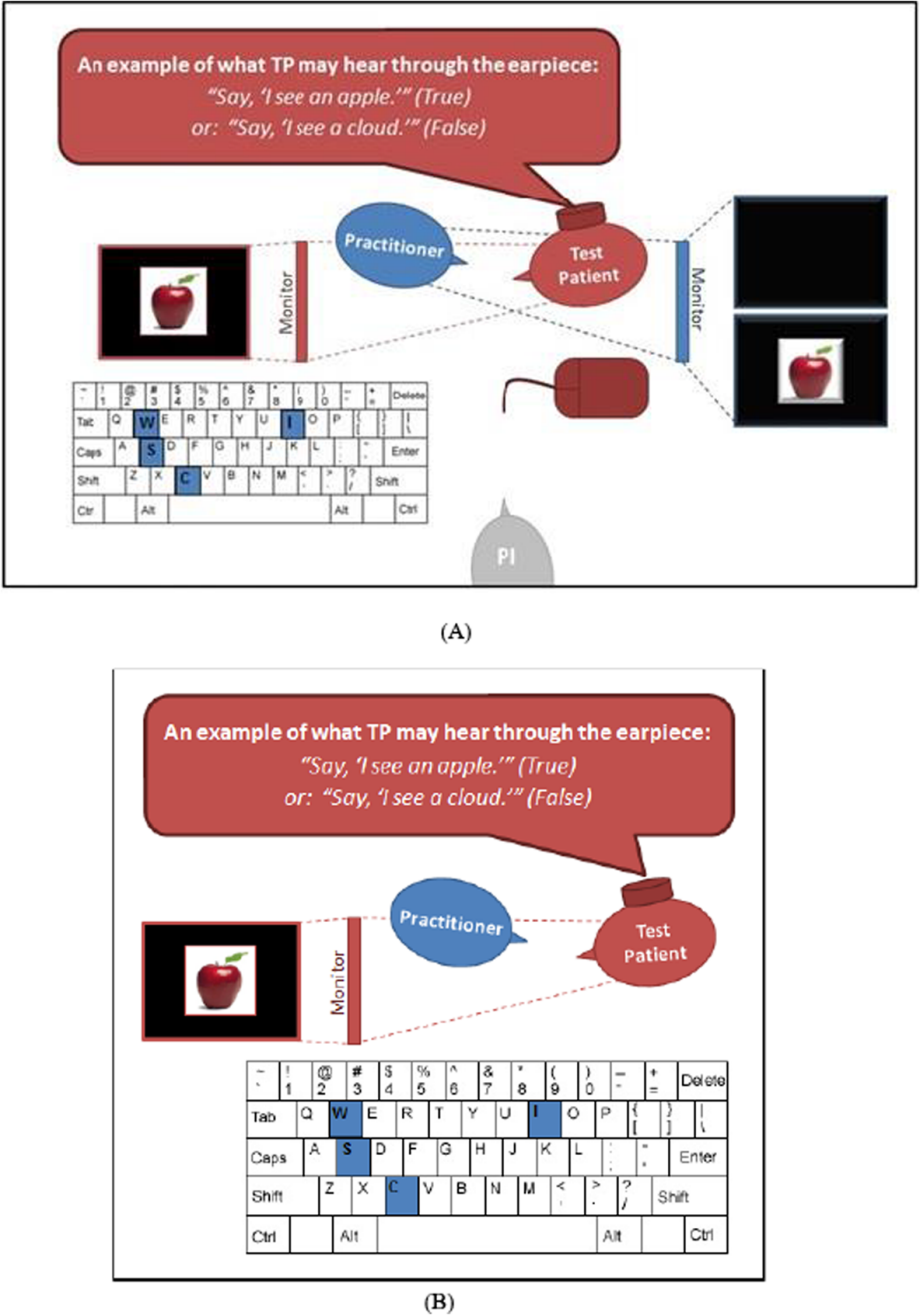
Testing Scenario Layout: **a** Experiment 1. The Test Patient (TP; *red*) viewed a monitor (also *red*) which the Practitioner could not see, had an ear piece in his ear through which he received instructions, and used a mouse to advance his computer to the next picture/statement. The Practitioner (*blue*) also viewed a monitor (also blue) which the Test Patient could not see and entered his results on a keyboard. Note that the Practitioner was presented with the same picture as the Test Patient or a blank, black screen. Also note that the Principal Investigator (PI) was present in the room and observing during all assessments. **b** Experiment 2. The TP (*red*) viewed a monitor (also *red*) which the Practitioner could not see, had an ear piece in his ear through which he received instructions, and used a mouse to advance his computer to the next picture/statement. In this Experiment, the Practitioner did not view a monitor, and still entered his results on a keyboard. Note that the Principal Investigator (PI) was absent during this Experiment

The methodology of this study followed that of Experiment 1, with the following exceptions: (1) practitioners in this study were invariably blind to the verity of the TPs’ statements; (2) the pairs were alone in the room for all tests; (3) practitioners rated their subjective state anxiety prior to testing; and (4) the prevalence of lies was fixed at 0.50. See Additional file 6: Figure S2 for the participant flow diagram, and Fig. 2 for an example of the testing layout.

### Statistical methods

For each practitioner-TP pair, accuracy of MRT was defined as the overall fraction correct when using MRT with the practitioner blinded to the true result. For Experiment 1, pilot data was used to estimate a sample size. In the pilot, MRT accuracy was found to be 67.7% correct (95% CI 52.6% to 82.8%). Based on this statistic and using a 95% confidence interval and 80% power, it was estimated that a study of 48 practitioner-TP pairs would be adequate to demonstrate whether trained practitioners can use MRT to distinguish a lie from a truth.

We report mean accuracy of MRT across all patients, with 95% confidence intervals. Accuracy of intuition was defined and reported similarly. Prior to analysis, normality assumptions were checked graphically (data not shown). Paired t-tests were used to test the null hypothesis that the mean difference in accuracy between MRT and intuition and zero. Secondary outcomes sensitivity, specificity, positive predictive value (PPV) and negative predictive value (NPV) were reported and analysed similarly. Linear regression was used to test for associations between accuracy and covariates: age, gender, profession, years in practice, current practice status, length and degree of MRT experience, types of MRT techniques trained in, left- or right-handedness, self-reported score for confidence in using MRT, and self-reported degree of testing anxiety. All analyses were restricted to tests for which the practitioner was blinded to the true answer. Analyses were conducted in Stata 12.1 (StataCorp LP, College Station, Texas).

## Results

### Experiment 1

#### Participants

Forty-eight unique practitioner-TP pairs were enrolled between June 2010 and October 2011, in the United Kingdom and the United States. Four volunteer practitioners did not meet the age criteria (i.e. they were aged > 65 years), one lacked fluency in English and one was hearing impaired. Of the 48 TPs enrolled, 31 were female and 17, male, and their mean (Standard Deviation, SD) age was 39.0 (11.4) years. In the sample of practitioners, there were 16 males and 32 females, the mean (SD) age was 49.3 (12.0) years, the median (Interquartile Range, IQR) number of years in practice was 11.5 (7.3 to 20.8) years, the median (IQR) years of MRT experience was 11.5 (5.3 to 17.3) years, and the median (IQR) hours of performing MRT/day was 2.9 (1.0 to 6.0) hours. The mean (SD) self-ranked MRT Expertise was found to be 3.1 (0.2) on a scale of 0 to 4. For a summary of practitioner demographics, see Additional file 7: Table S1.

#### Test results

The primary outcome, MRT accuracy (i.e. overall fraction correct) during tests when the practitioner was blinded to the truth of the statement, ranged between 0.400 and 0.917, and the mean (95% Confidence Interval, CI) was 0.659 (0.623 to 0.695). The accuracy of intuition for detecting lies during tests when the practitioner was blinded ranged between 0.238 and 0.636, and the mean (95% CI) was 0.474 (0.449 to 0.500). The mean accuracy of MRT for detecting lies was significantly greater than mean accuracy of intuition for detecting lies (*p* = 0.01; see Table 1). The mean accuracy of MRT for detecting lies was also significantly greater than 0.5 (i.e. *chance*; *p* < 0.01). There was no significant correlation between practitioners’ accuracy using MRT to detect lies and their accuracy using their intuition to detect lies (*r* = -0.03, *p* = 0.86, 95% CI -0.31 to 0.26).

**Table 1:** Comparing mean accuracy statistics (with 95% Confidence Intervals) of MRT and Intuition, for Experiments 1 and 2

	Experiment 1					Experiment 2				
	(*n* = 48)					(*n* = 20)				
	MRT		Intuition			MRT		Intuition		
	Mean	95% CI	Mean	95% CI	*p*-value	Mean	95% CI	Mean	95% CI	*p*-value
Overall Fraction Correct	0.659	0.623 - 0.695	0.474	0.449 - 0.500	<0.01*	0.594	0.541 - 0.647	0.514	0.483 - 0.544	0.01*
Sensitivity	0.568	0.504 - 0.633	0.429	0.374 - 0.484	<0.01*	0.583	0.534 - 0.631	0.603	0.555 - 0.650	0.61
Specificity	0.734	0.687 - 0.782	0.479	0.416 - 0.542	<0.01*	0.613	0.553 - 0.673	0.494	0.427 - 0.561	0.01
Positive Predictive Value	0.663	0.607 - 0.718	0.527	0.460 - 0.594	<0.01*	0.685	0.616 - 0.754	0.603	0.555 - 0.650	0.06
Negative Predictive Value	0.667	0.625 - 0.708	0.392	0.335 - 0.448	<0.01*	0.503	0.421 - 0.584	0.425	0.356 - 0.494	0.07

*MRT* muscle response testing, *CI* confidence interval; *reached significance

The mean (95% CI) sensitivity of MRT for detecting lies was 0.568 (0.504 to 0.633) and the mean (95% CI) specificity (i.e. accuracy for identifying truth) was 0.734 (0.687 to 0.782), while the mean (95% CI) PPV for MRT was 0.663 (0.607 to 0.718) and the mean (95% CI) NPV for MRT was 0.667 (0.625 to 0.708). See Table 1, which also contains the same statistics for the intuition condition. The 2x2 tables for each practitioner-TP pair can be found in Additional file 7: Table S2.

Table 2 shows analyses of accuracy by practitioner characteristics, and excludes two practitioners who did not complete the questionnaire. Mean MRT accuracy (95% CI) by practitioner profession for the 20 chiropractors who participated was 0.670 (0.611 to 0.729), and for non-chiropractors, 0.642 (0.593 to 0.691), which were not significantly different (*p* = 0.45) in MRT accuracy. Mean accuracy (95% CI) for those in full-time practice (*n* = 26) was 0.663 (0.612 to 0.715), part-time practice (*n* = 13), 0.682 (0.618 to 0.746), and not practising (*n* = 7), 0.569 (0.465 to 0.673), which also were not significantly different (*p* = 0.45) in MRT accuracy. Mean MRT accuracy (95% CI) of those practitioners who ranked themselves in the highest category for expertise as “Expert” muscle testers (level 4 of 4; *n* = 15) was 0.682 (0.617 to 0.747), of those who ranked themselves in the second highest category (level 3 of 4; *n* = 19) was 0.666 (0.605 to 0.728), and of those who ranked themselves in lower categories (levels 1 or 2 of 4; *n* = 12), 0.600 (0.528 to 0.672), with *p* = 0.35 for difference between expertise levels. Table 2 also compares the mean accuracies in practitioner-TP pairs in which the TP reported guessing the paradigm with those whose TPs did not. When the TP reported guessing the paradigm (*n* = 21), the mean accuracy of MRT was 0.661 (95% CI 0.591 to 0.730), and for those pairs in which the TP did not report guessing the paradigm (*n* = 27), the mean accuracy of MRT was 0.649 (95% CI 0.610 to 0.688), and there was no significant difference between these two groups (*p* = 0.38) in MRT accuracy. See Table 2.

**Table 2:** The influence on various categorical participant characteristics on MRT Accuracy. (1) Practitioner profession, (2) Practitioner’s practising status, (3) Practitioner’s self-ranked MRT expertise,^c^ and (4) If the test patient reported guessing the paradigm

		MRT ACCURACY									
		(1)		(2)			(3)			(4)	
		Practitioner profession		Practitioner practising status			Self-ranked MRT experise			TP reported guessing the paradigm?	
		Chiropractors	All others	Full Time	Part Time	Not Practising	4	3	1 or 2	Yes	No
		(*n* = 20)	c	(*n* = 26)	(*n* = 13)	(*n* = 7)	(*n* = 15)	(*n* = 19)	(*n* = 12)	(*n* = 21)	(*n* = 27)
Experiment 1	Mean	0.670	0.642	0.663	0.682	0.569	0.682	0.666	0.600	0.661	0.649
	95% CI	0.611 - 0.729	0.593 - 0.691	0.612 - 0.715	0.618 - 0.746	0.465 - 0.673	0.617 - 0.747	0.605 - 0.728	0.528 - 0.672	0.591 - 0.730	0.610 - 0.688
	*p*-value	0.45^a^		0.13^b^			0.35^b^			0.38	
Experiment 2		Chiropractors	All other	Full Time	Part Time	Not Practising	4	3	2	Yes	No
		(*n* = 14)	(*n* = 6)	(*n* = 14)	(*n* = 4)	(*n* = 2)	(*n* = 7)	(*n* = 10)	(*n* = 3)	(*n* = 6)	(*n* = 14)
	Mean	0.607	0.563	0.561	0.706	0.600	0.611	0.590	0.567	0.621	0.582
	95% CI	0.535 - 0.679	0.478 - 0.647	0.504 - 0.618	0.508 - 0.905	0.000 - 1.000	0.470 - 0.751	0.518 - 0.662	0.387 - 0.746	0.507 - 0.735	0.515 - 0.650
	*p*-value	0.36		0.07			0.86			0.49	

*MRT* muscle response testing, *CI* confidence interval; ^a^ *t*-test result; ^b^ANOVA result; ^c^Practitioners were asked to rank their own MRT ability from 0 (“None”) to 4 (“Expert”)

There was no obvious trend in accuracy over time during the course of experiments (see Additional file 7: Table S3 and Additional file 8: Figure S3). A *post hoc* analysis found no significant difference between results in a location which was particularly noisy compared to other study sites (*p* = 0.46). With the exception of shoulder muscle fatigue (*n* = 7 out of 96 participants), no adverse events were reported during testing.

### Experiment 2

#### Participants

Twenty unique practitioner-TP pairs were enrolled between July and November 2011, in the United Kingdom and the United States, including 13 female and 7 male practitioners, and 8 female and 12 male TPs. The mean (SD) age for practitioners was 49.3 (12.0) years, and for TPs, 40.8 (12.8) years. Of the 20 practitioners enrolled there were 14 chiropractors, 2 mental health professionals, 1 acupuncturist, and 3 other health professionals. Fourteen practitioners were in full-time practice, 4 were in part-time practice, and 2 were not currently practising. The practitioners’ median (IQR) number of years in practice was 18.0 (17.0) years, the median (IQR) years of MRT experience was 14.0 (16.0), and the median (IQR) hours of performing MRT/day was 4.0 (4.0). The mean (SD) self-ranked MRT Expertise was found to be 3.2 (0.7) on a scale of 0 to 4. For a summary of practitioner demographics, see Additional file 7: Table S1.

#### Test results

In Experiment 2, the mean (95% CI) MRT accuracy (i.e. overall fraction correct) for detecting lies was 0.594 (0.541 to 0.647), and ranged between 0.425 and 0.825. The mean (95% CI) accuracy when using intuition for detecting lies was 0.514 (0.483 to 0.544), and ranged between 0.375 and 0.625. The mean accuracy when using MRT for detecting lies was significantly greater than when using intuition (*p* = 0.01; see Table 1). The mean accuracy of MRT was also significantly greater than 0.5 (i.e. *chance*; *p* < 0.01). There was no significant correlation between practitioners’ accuracy using MRT for detecting lies and their accuracy using their intuition (*r* = 0.07, *p* = 0.77, 95% CI -0.38 to 0.50).

The mean (95% CI) sensitivity for MRT for detecting lies was 0.583 (0.534 to 0.631) and the mean (95% CI) specificity (i.e. the accuracy of MRT for detecting truth) was 0.631 (0.553 to 0.673), while the mean (95% CI) PPV for MRT was 0.685 (0.616 to 0.754) and the mean (95% CI) NPV for MRT was 0.503 (0.421 to 0.584). See Table 1, which also contains the same statistics for the intuition condition. The 2x2 tables for each practitioner-TP pair can be found in Additional file 7: Table S4.

Analyses of MRT accuracy by practitioner characteristics can be found in Table 2. The mean MRT accuracy (0.607; 95% CI 0.535 to 0.679) for the 14 chiropractors who participated was not significantly different (*p* = 0.36) from the mean MRT accuracy (0.563; 95% CI 0.478 to 0.647) for the 6 non-chiropractors. The mean accuracy (95% CI) for those in full-time practice (*n* = 14) was 0.561 (0.504 to 0.618), part-time practice (*n* = 4), 0.706 (0.508 to 0.905), and not practising (*n* = 2), 0.600 (0.000 to 1.000), and there was no significant difference between these groups (*p* = 0.07) in MRT accuracy. The mean MRT accuracy (95% CI) of those practitioners who ranked themselves in the highest category for expertise (i.e. “Expert”) in muscle testing (level 4 of 4; *n* = 7) was 0.611 (0.470 to 0.751), of those who ranked themselves in the second highest category (level 3 of 4; *n* = 10) was 0.590 (0.518 to 0.662), and of those who ranked themselves in lower categories (levels 1 or 2 of 4; *n* = 3), 0.567 (0.387 to 0.746), and there was no significant difference between these groups (*p* = 0.86) in MRT accuracy. Table 2 also compares the mean accuracies in practitioner-TP pairs in which the TP reported guessing the paradigm with those which the TPs did not. When the TP reported guessing the paradigm (*n* = 6), the mean accuracy of MRT was 0.621 (95% CI 0.507 to 0.735), and for those pairs which the TP did not report guessing the paradigm (*n* = 14), the mean accuracy of MRT was 0.582 (95% CI 0.515 to 0.650), and no significant difference was found between these two groups (*p* = 0.49) in MRT accuracy. See Table 2. Similar to Experiment 1, with the exception of muscle fatigue (*n* = 4 out of 40 participants), no adverse events were reported during testing.

## Discussion

### Statement of the principal findings

Muscle response testing (MRT) used for distinguishing false from true spoken statements was consistently found to be more accurate than would be expected by chance. It was also better than intuition employed by the same practitioner, indicating that success was due to the muscle testing component rather than, for example, body language or voice qualities. These studies provide one step toward proof of concept for this application of MRT. They also demonstrate that scientific methods, including blinding and randomisation, can be used in the assessment of tests used by complementary and alternative medicine practitioners, such as MRT.

All analyses presented here were for tests for which the practitioner was blinded to the true answer; results for test in which the practitioner was not blinded, and for a further experiment in which the practitioner was actively deceived, have been reported elsewhere [25].

### Strengths and limitations

These studies did not standardise MRT methods, for instance, by utilising force plates to monitor pressure. The strength of this approach is that the MRT performed in these studies is comparable to that used by these practitioners in their clinical practice. Supporting this decision, previous studies using force plates showed a distinct difference between muscles labelled “strong” and “weak” [12, 26, 27], making their use in these studies redundant. Other strengths include the high degree of blinding and well-defined reference standard and target condition. However, the statements used as reference standard were not designed to be representative of those that might be of interest in clinical practice. We did not evaluate MRT against other widely-used methods of ‘lie detection’, such as polygraph [28]. Other proposed applications of MRT, such as for the diagnosis of a food allergy [9, 29] or the need for a nutritional supplement [30] or to assess athletic performance [31–33], are beyond the scope of our studies.

Although practitioners were blinded to veracity of the statement, test patients necessarily were not. However, there was no significant difference in results between pairs in which the test patient guessed the paradigm (that strong response indicated a true statement) and other pairs, making it less likely that results are explained by test patients consciously or nonconsciously biasing the test. In addition, these studies would have been strengthened if the order of the blocks were randomised, with some pairs starting with MRT and other pairs starting with Intuition.

### Strengths and weaknesses in relation to other studies

One other published study attempted to estimate the accuracy of MRT to distinguish truth from lies [12]. However, in this study, specific characteristics about the practitioners performing the MRT are unclear, such as how many were enrolled, how they were recruited, the inclusion/exclusion criteria, and the degree of practitioner blinding [12]. These important features may have limited the usefulness of this study. Another study assessed practitioners ability to distinguish weak from strong responses, but did not examine whether this was correlated with true and false, therefore a practical comparison is difficult [34].

### Implications for clinical practice and future research

We have provided one step toward proof of concept that MRT is better than chance alone at distinguishing true from false statements. However, the statements studied here are not necessarily typical of those relevant in practice, and the average accuracy, though significantly better than chance or intuition, was found to be 60 to 70%. The accuracy necessary for improving patient outcomes in practice is unclear and may depend upon factors beyond the scope of our studies [35, 36]. The variation in accuracy between those pairs assessed may suggest the existence of practitioner characteristics that influence accuracy; if so, and if these are modifiable characteristics, it may be possible to develop protocols for consistently high accuracy.

We have demonstrated that scientifically rigorous methods, including blinding, randomness, use of a comparator, and formal statistical analysis, can be applied constructively to MRT research. Research is needed to assess the usefulness of MRT for detecting other commonly-used target conditions, such as the need for nutritional supplementation [13, 20, 36, 37] or in the identification of an allergy or hypersensitivity or toxicity [3, 9, 11, 22, 38–45].

Future research in the diagnostic usefulness of MRT should employ rigorous methods, including: (1) a clear and specific research objective, (2) a well-defined target condition, (3) explicit outcomes that are easy to interpret, (4) an appropriate sample of the target population (who were objectively selected), (5) an objective reference standard, (6) an adequate sample size, and (7) appropriate blinding [36].

Finally, due to its widespread use [5], MRT’s true clinical value must be explored [38, 46–50]. Toward this end, the efficacy of MRT technique systems must be investigated via rigorously-designed randomised, controlled trials (RCTs). For example, future researchers may want to explore the effectiveness of alternative stress reduction techniques which use MRT, such as HeartSpeak, for such conditions as depression or panic attacks, compared to traditional psychological approaches, such as cognitive behavioural therapy.

## Conclusion

Muscle response testing (MRT) has repeatedly been found to be significantly more accurate than both intuition and chance, for one application of this common assessment method: distinguishing lies from truths. No test is perfect: 100% accurate, easy to use, risk-free and low cost [36, 41]. However, these results are encouraging. It is hoped that this report will encourage further research on the clinical utility of MRT.

## Additional files

Additional file 3: Table S6. STARD checklist for reporting of studies of diagnostic accuracy: *Experiment 1*. (DOCX 19 kb)
Additional file 4: Table S7. STARD checklist for reporting of studies of diagnostic accuracy: *Experiment 2*. (DOCX 19 kb)
Additional file 5: Figure S1. Participant Flow Diagram - Experiment 1. (JPG 56 kb)
Additional file 6: Figure S2. Participant Flow Diagram - Experiment 2. (JPG 59 kb)
Additional file 7: Table S1. Demographics of Practitioners - Experiments 1 & 2. **Table S2.** 2x2 Table for MRT for each Pair (*n*=48) in Experiment 1. **Table S3.** Correlations (r) with *p*-values among MRT. **Table S4.** 2x2 Tables for MRT for each Pair in Experiment 2 (*n*=20). **Table S5.** Correlations (r) among MRT Accuracy and Practitioner haracteristics for Experiments 1 & 2. p(2-tailed)<0.05. (XLS 75 kb)
Additional file 8: Figure S3. kMMT Accuracy by Block with 95% Confidence Intervals. (DOCX 18 kb)

## Abbreviations

CI: Confidence interval
FN: False negatives
FP: False positives
IQR: Interquartile range
MMT: Manual muscle testing
MRT: Muscle response testing
NPV: Negative predictive value
PPV: Positive predictive value
SD: Standard deviation
STARD: Standards for the Reporting of Diagnostic Test Accuracy Studies
TP: Test patient
