# Supplementary figures and images for: Estimating the accuracy of muscle response testing: two randomised-order blinded studies
Source: BMC Complement Altern Med. 2016 Nov 30;16:492. doi: 10.1186/s12906-016-1416-2 (PMC5131520; doi:10.1186/s12906-016-1416-2)

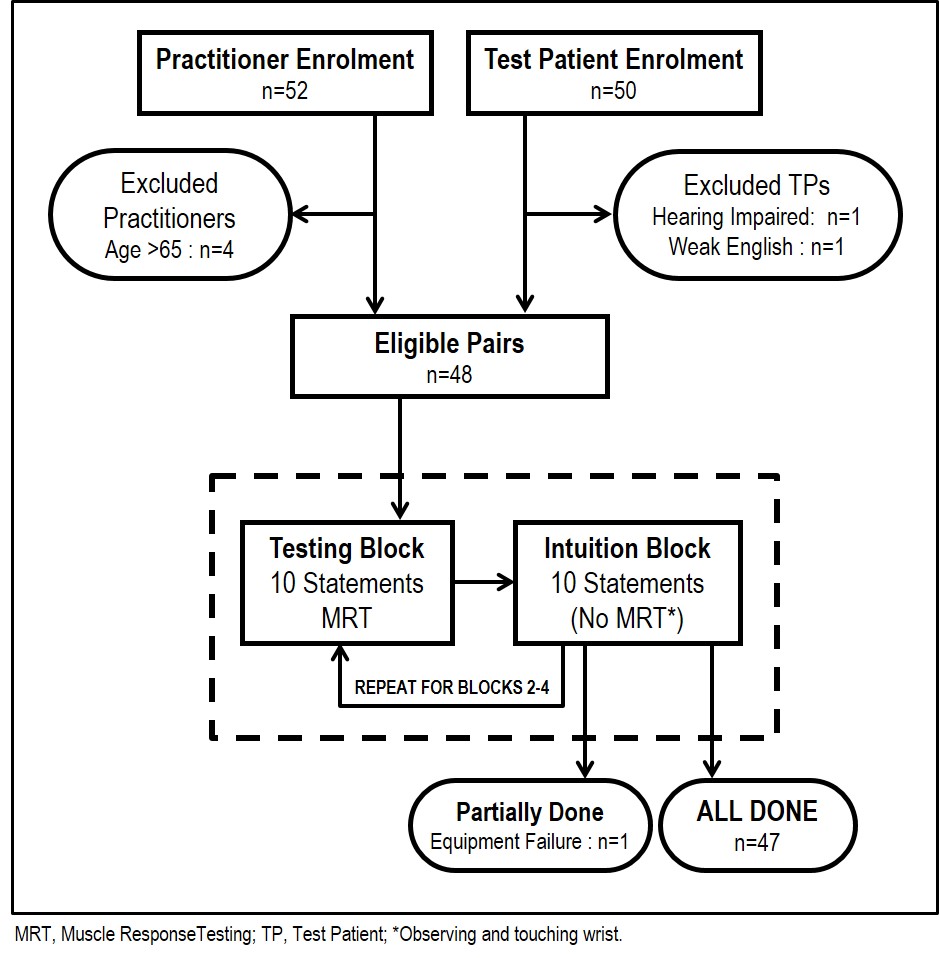

Supplement: Additional file 5: Figure S1. — Participant Flow Diagram - Experiment 1. (JPG 56 kb) [file 12906_2016_1416_MOESM5_ESM.jpg]

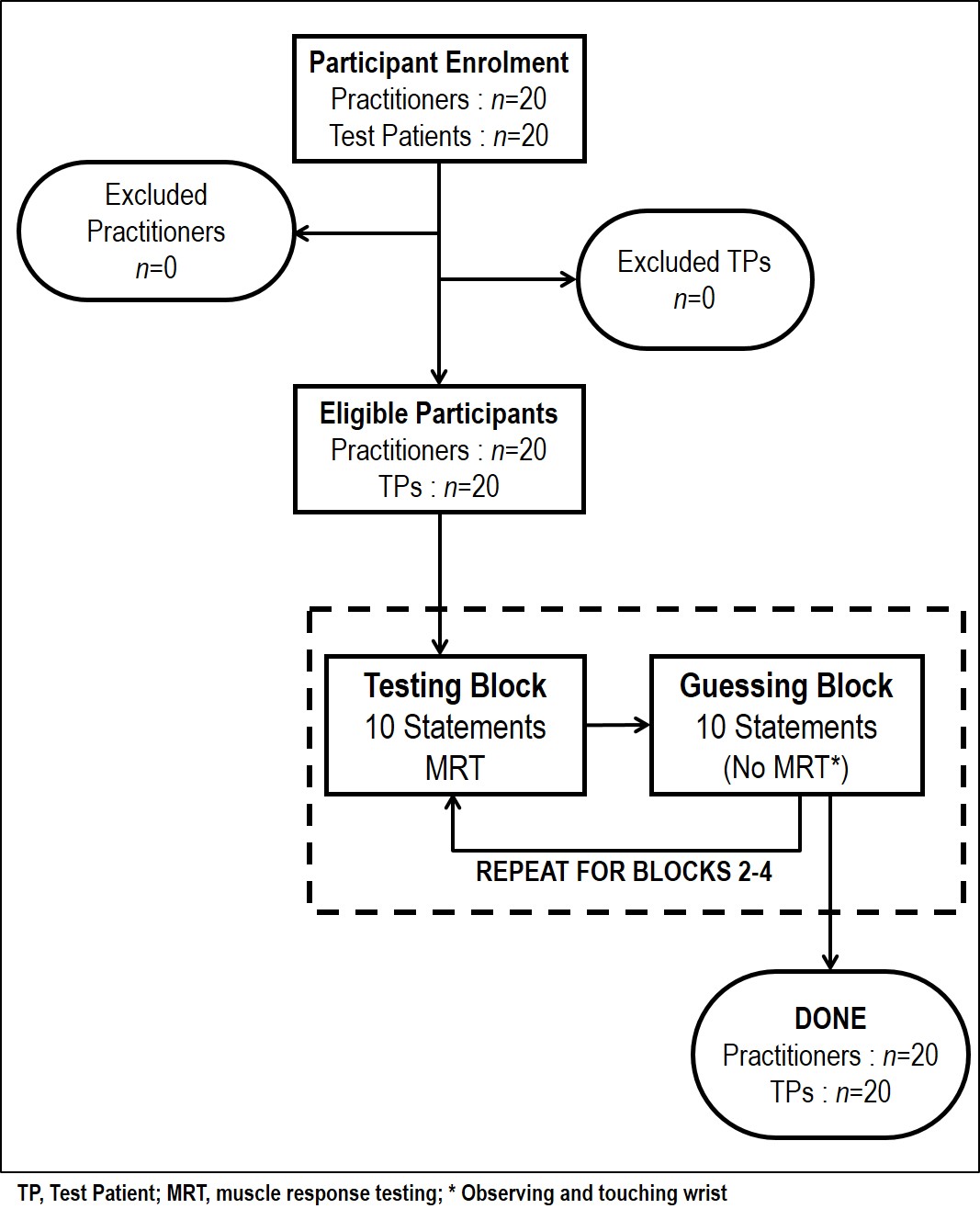

Supplement: Additional file 6: Figure S2. — Participant Flow Diagram - Experiment 2. (JPG 59 kb) [file 12906_2016_1416_MOESM6_ESM.jpg]
