# Supplementary material for: Estimating the accuracy of muscle response testing: two randomised-order blinded studies
Source: BMC Complement Altern Med. 2016 Nov 30;16:492. doi: 10.1186/s12906-016-1416-2 (PMC5131520; doi:10.1186/s12906-016-1416-2)
Supplement: Additional file 8: Figure S3. — kMMT Accuracy by Block with 95% Confidence Intervals. (DOCX 18 kb) [file 12906_2016_1416_MOESM8_ESM.docx]

**SUPPLEMENTARY FIGURE S3 – kMMT Accuracy by Block with 95% Confidence Intervals.**
